# Polymeric Cups for Cavitation-mediated Delivery of Oncolytic Vaccinia Virus

**DOI:** 10.1038/mt.2016.139

**Published:** 2016-08-09

**Authors:** Rachel Myers, Christian Coviello, Philippe Erbs, Johann Foloppe, Cliff Rowe, James Kwan, Calum Crake, Seán Finn, Edward Jackson, Jean-Marc Balloul, Colin Story, Constantin Coussios, Robert Carlisle

**Affiliations:** 1 Department of Engineering Science, University of Oxford, BUBBL, IBME, Oxford, UK; 2 OxSonics Ltd, The Magdalen Centre, Oxford, UK; 3 Transgene SA, Boulevard Gonthier d'Andernach, Illkirch-Graffenstaden, France

## Abstract

Oncolytic viruses (OV) could become the most powerful and selective cancer therapies. However, the limited transport of OV into and throughout tumors following intravenous injection means their clinical administration is often restricted to direct intratumoral dosing. Application of physical stimuli, such as focused ultrasound, offers a means of achieving enhanced mass transport. In particular, shockwaves and microstreaming resulting from the instigation of an ultrasound-induced event known as inertial cavitation can propel OV hundreds of microns. We have recently developed a polymeric cup formulation which, when delivered intravenously, provides the nuclei for instigation of sustained inertial cavitation events within tumors. Here we report that exposure of tumors to focused ultrasound after intravenous coinjection of cups and oncolytic vaccinia virus , leads to substantial and significant increases in activity. When cavitation was instigated within SKOV-3 or HepG2 xenografts, reporter gene expression from vaccinia virus was enhanced 1,000-fold (*P* < 0.0001) or 10,000-fold (*P* < 0.001), respectively. Similar increases in the number of vaccinia virus genomes recovered from tumors were also observed. In survival studies, the application of cup mediated cavitation to a vaccinia virus expressing a prodrug converting enzyme provided significant (*P* < 0.05) retardation of tumor growth. This technology could improve the clinical utility of all biological therapeutics including OV.

## Introduction

Oncolytic viruses (OV) represent a powerful platform for achieving cancer therapy due to their tumor-selective self-amplification and their ability to provide expression of therapeutic proteins from within tumors.^1,2^ The next decade is likely to see a wide range of these agents continuing their progression through the clinical testing pathway both in combination with conventional anticancer strategies^3^ and immuno-oncology approaches.^4^ However, the poor delivery of OV into and throughout target tumors following systemic administration means that, to date, the majority of clinical applications are reliant on direct intratumoral injection, a route which is inefficient^5^ and restricts the type of cancer which can be treated. In response, several methods have been employed to permit the intravenous delivery of OV, and thereby broaden their potential clinical utility. Indeed, recent studies have addressed their rapid neutralization in the bloodstream^6,7,8^ as well as their limited transfer from the bloodstream into tumors.^9^ Notably, the activity of an OV within xenograft tumors was enhanced up to 50-fold by coinjection of SonoVue (SV), an ultrasound (US) contrast agent, and simultaneous exposure of the tumors to focused US.^10^ Such delivery was mediated by the SV responding to US and acting as a nuclei for the instigation of inertial cavitation events, which in turn caused the microstreaming and shockwaves responsible for propelling the OV into and throughout tumors.^11^ Moreover, increasing the density of the virus further enhanced this effect.^12^ However, whilst SV represents a useful tool to demonstrate that US induced inertial cavitation can provide substantial enhancement of OV tumor delivery, it has poor clinical translatability due to its rapid destruction and 1–10 µm diameter. We have recently described the formulation of a novel polymeric cup (“cups”) cavitation inducing agent which measures <500 nm.^13^ This cups formulation provides a level of cavitation from within tumors which is more sustained than that achieved with SV. Furthermore, whereas the micron size of SV spatially restricts its impact to the tumor vasculature, cups can self-propel through the tumor vasculature and continue to assist transport within.^13,14^ We use oncolytic vaccinia virus (VV) to demonstrate that this technology can achieve dramatic increases in the efficiency of virus delivery and tumor infection which, ultimately, leads to improvements in tumor growth retardation and overall survival.

## Results

### Impact of cup nucleated cavitation on VV delivery

Our previous cavitation mediated delivery studies have only utilized nonenveloped viruses^10^ and so tests were performed to characterize the stability of VV to cavitation events (see **Supplementary Figure S1a**). 1 × 10^6^ plaque forming units (pfu) of a luciferase expressing VV (VVluc) were exposed to US. No decrease in the ability of the VVluc to infect CT-26 cells and produce luciferase transgene resulted from the exposure of VVluc to cups mediated cavitation.

**Figure 1:**
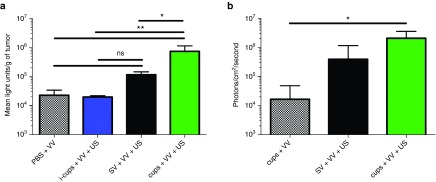
***In vivo* infectivity of vaccinia virus (VV) delivered using SonoVue (SV) or polymeric cup (“cups”) nucleated cavitation.** A dose of 1 × 10^5^ luciferase expressing VV was mixed with inactive cups (i-cups), SV or cups and injected into (**a**) Balb/c mice bearing CT-26 tumors. The tumors were exposed to ultrasound (US) (see Methods for parameters) and 48 hours later tumors were excised, homogenized and luciferase expression quantified (see Methods). (**b**) CD-1 nude mice bearing HepG2 tumors. The tumors were exposed to US and 5 days later luciferase expression was assessed by an *in vivo* imaging system (IVIS) (see Methods). *n* = 4, SD shown, significant differences (*P < 0.05, **P < 0.01, ns, nonsignificant *P* > 0.05) detected by one-way analysis of variance (ANOVA) with Tukey compare all columns post-test.

The benefit of the use of cups compared with SV was demonstrated by comparing their impact on the delivery of luciferase expressing VVluc to CT26 tumors in Balb/C mice, a model in which replication is suboptimal (**Figure 1a**). Mice were sacrificed and tumors analyzed 48 hours after treatment and even at this early time-point a >fivefold increase in luciferase expression was evident for SV + VVluc + US treated mice compared with the VV alone group. Although large, this effect did not reach significance (in line with a previous study using Adenovirus and SV, where SV produced impressive yet variable results^9^). Notably, cups + VVluc + US provided an even greater enhancement of delivery, achieving levels of luciferase which were >45-fold greater than VV alone and sixfold greater than achieved using SV + VVluc + US (738,450 versus 115,855 light units/g tumor, *P* < 0.01), (**Figure 1a**). The administration of VVluc with inactivated cups (i-cups), cups which were formulated to be unresponsive to US, and US exposure provided no enhancement of reporter gene expression compared with the delivery of VVluc alone (light units/g tumor = 19,773 versus 22,760 *P* > 0.05). This demonstrates that neither cups alone nor US alone is a sufficient condition to enhance delivery of the VV. Passive acoustic mapping performed during exposure showed that in the presence of i-cups, the US parameters used here were insufficient to create cavitation within the tumor. Furthermore, such mapping demonstrated the superior maintenance of cavitation signal achieved with cups versus SV (see **Supplementary Figure S1b**). To probe the impact of improved delivery on the ability of VV to replicate and spread over prolonged durations these experiments were extended with the use of a CD-1 nude mouse with HepG2 tumors, a model which is more amenable to supporting VV infection. In this way, the two studies in **Figure 1** allowed assessment of the impact of cavitation on both initial delivery (CT26 cells) and subsequent spread (HepG2 cells). Notably, 5 days after treatment, mice with HepG2 tumors dosed with cups + VV + US provided more than 125-fold greater luminescent signal compared with those dosed with cups + VV alone (*P* < 0.05) and sixfold increase over those dosed with SV + VV + US (**Figure 1b**). The images of these mice (shown in **Supplementary Figure S2**) also serve to demonstrate the greater reproducibility of delivery achieved using cups rather than SV.

### Impact of cup nucleated cavitation on VV infection of tumors

**Figure 2:**
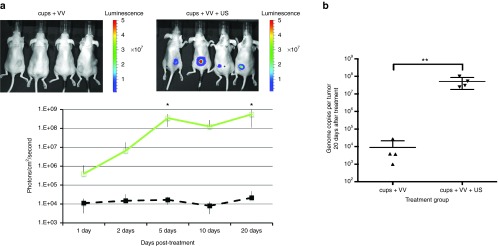
***In vivo* infectivity of vaccinia virus (VV) delivered using polymeric cup (“cups”) nucleated cavitation to HepG2 tumours.** A dose of 1 × 10^5^ luciferase expressing VV was mixed with cups and injected into mice and their tumors exposed to ultrasound (US) (see methods for parameters). Passive acoustic mapping confirmed the absence or presence of cavitation within the tumor. (**a**) Luciferase expression was assessed by an *in vivo* imaging system (IVIS) imaging at intervals over the next 20 days (see Methods, inset images show luciferase expression of tumours at day 10). Green line = cups + VV + US, black dashed line = cups + VV. (**b**) VV genome copy number within the tumors of the mice was measured at sacrifice on day 20 (see methods). *n* = 4, SD shown, significant differences (*P < 0.05, **P < 0.01) detected by analysis of variance (ANOVA) with Bonferroni compare all columns post-test.

Having demonstrated the advantage of the use of cups over SV, the SV group could now be excluded from further experiments. When CD1 nude mice bearing xenograft HepG2 tumors were coinjected with 2.5 mg of cups and just 1 × 10^5^ pfu of VVluc, very low levels (~1 × 10^4^ photons/second/cm^2^) of luciferase expression were detected by an *in vivo* imaging system (IVIS) at 24 hours (**Figure 2a**). This level did not substantially increase over the subsequent 20 days, with only one mouse of four showing levels exceeding 1 × 10^5^ photons/second/cm^2^. In contrast, when the exact same procedure was performed while the tumor was exposed to US (see Methods for details) luciferase expression reached 3.1 × 10^5^ photons/second/cm^2^ by 24 hours and 3.5 × 10^8^ by 10 days, this level was maintained in all these mice until sacrifice at 20 days. Mapping of the cavitation within tumors allowed real-time validation of the success of cavitation instigation and confirmed the presence of cups within tumors. Quantitative polymerase chain reaction of tumors rescued following sacrifice at day 20 (see Methods) confirmed the benefit of cup mediated cavitation enhanced delivery (**Figure 2b**). In the tumors of mice treated with cups + VV without US, negligible levels of VV DNA were recovered. In contrast tumors from cups + VV + US treated mice contained nearly 1 × 10^8^ VV genome copies, representing ~1,000-fold increase compared with the original IV injected dose and a 10,000-fold increase compared with tumors of mice treated with cups but no US. Meanwhile, VV expression in the liver remained equally negligible in the cups + VV group and the cups + VV + US group as evident from IVIS imaging (**Figure 2**) and from quantitative polymerase chain reaction analysis following sacrifice at day 20 (see **Supplementary Figure S3**).

When these experiments were repeated in mice bearing SKOV-3 tumors the same pattern was observed (see **Supplementary Figure S4**), with the production of cavitation within the tumor again correlating with 1,000-fold increase in luciferase expression and genome copy number at 20 days (see **Supplementary Figure S4a, b**).

### Impact of cup nucleated cavitation on VV retardation of tumor growth

**Figure 3:**
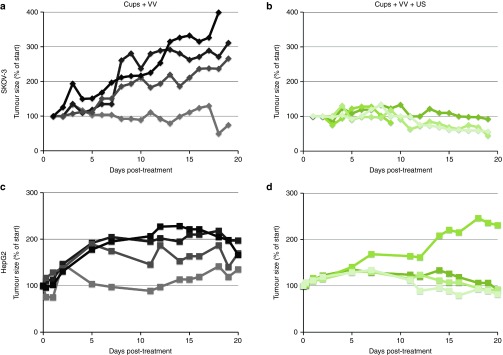
**Retardation of SKOV-3 or HepG2 tumor growth in mice dosed IV with 1 × 10^6^ copies of vaccinia virus (VV).** A dose of 1 × 10^6^ luciferase expressing VV was injected IV with cups with or without the application of ultrasound (US) (see Methods). Tumor size was assessed by caliper measurements. Growth profile shown for each individual tumor. *n* = 4. Panels **a** and **b** present SKOV-3 tumors, **c** and **d** present HepG2. Panels **b** and **d** are mice treated with cups + VV + US.

HepG2 tumor growth was not affected when mice were dosed with just 1 × 10^5^ VVluc, regardless of the delivery enhancement provided by cups and US (see **Supplementary Figure S5**). It is hypothesized that, despite the substantial and significant increase in VVluc concentration in the tumor achieved by the cup and US treatment, the VVluc dose in the tumor is still below the efficacious concentration. Approaches to overcome this therapeutic threshold include increasing the dose of VVluc used or arming the VV with a therapeutic transgene rather than the reporter gene luciferase. The first of these options was explored in **Figure 3**, where a dose of 10^6^ VVluc was used in combination with cups and US.

Retardation of HepG2 and SKOV-3 tumor growth was observed in mice treated with cups + VV + US compared with controls which received cups + VV but no US. In four out of four SKOV-3 tumors and in three out of four HepG2 tumors growth was controlled when cups + VV + US was used, whereas all but one tumor treated with just cups + VV showed continued growth. Notably the one tumor which did not respond to treatment in the cups + VV + US treated group showed the lowest cavitation response as detected by passive acoustic mapping, demonstrating the utility of such monitoring in identifying potential treatment failures. Analysis of mean data from these experiments demonstrated that although a significant (*P* < 0.05) impact on tumor growth in mice bearing SKOV-3 tumors was achieved with cups + VV + US, in mice bearing HepG2 tumors such significance was not reached (as a result of the mouse with “failed” levels of cavitation). This demonstrates the potential utility of this approach in enhancing treatment and the value of cavitation monitoring, but also emphasizes the need to test VV armed with therapeutic transgene rather than a reporter transgene.

Recent studies have demonstrated that the bloodstream neutralization of VV will rapidly reduce the active circulating dose of VV vectors.^7^ In the face of such a limitation, it is essential that that the small proportion of the dose that does remain bio-available is delivered into the tumor as effectively as possible and then has the maximal antitumor effect achievable.

**Figure 4:**
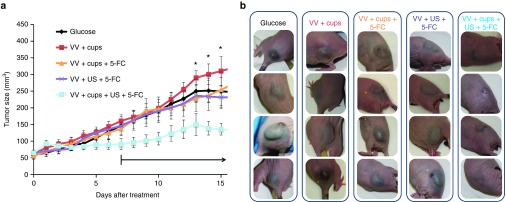
**Retardation of HepG2 tumor growth in mice dosed IV with 1 × 10**^**5**^ **copies of VVTK-RR-/FCU1 and dosed with 5-Fluorocytosine (5-FC).** A dose of 1 × 10^5^ vaccinia virus (VV), expressing an enzyme for the conversion of 5-FC to 5-fluorouracil (5-FU), was injected IV with or without the application of cups and ultrasound (US). After 7 days, 5-FC dosing commenced with daily s.c. injection of 100 µl of 12.5 mg/ml (black arrow). Tumor size was assessed by caliper measurements (**a**). *N* = 10, standard error of the mean shown, (*P < 0.05, by analysis of variance (ANOVA) with all group comparisons and Bonferroni post-test). Black arrow denotes commencement of daily dosing with 100 µl of 12.5 mg/ml 5-FC. (**b**) Representative mice from the study described in 4a were photographed 9 days after dosing with VV. Dramatic differences in the size and stiffness of the tumors were observed.

Enhancement of oncolytic effect can be provided by the expression of a range of proteins which may enhance spread,^15,16^ instigate an immune response,^17^ inhibit vascularisation^18^ or convert nontoxic prodrugs in to active metabolites.^19^ This prodrug converting enzyme approach has recently been further explored with the development of a VV (VVTK-RR-/FCU1) which encodes an enzyme which converts 5-Fluorocytosine (5-FC) into the antimetabolite chemotherapeutic 5-Fluorurocil (5-FU).^20,21^ In previous studies, when mice bearing xenograft tumors were dosed twice IV with VVTK-RR-/FCU1 at 1 × 10^6^ copies and 5-FC dosing commenced daily at 7 days, substantial tumor retardation was shown.^21^ The work reported in **Figure 2** using VVluc raised the possibility that an antitumor effect could even be achieved with VVTK-RR-/FCU1 at a dose of just 1 × 10^5^ copies, provided cups and US were used to enhance delivery. **Figure 4a** demonstrates that compared with all other control groups the use of cups + VVTK-RR-/FCU1 + US + 5-FC showed enhanced retardation of tumor growth (*P* < 0.05). Pictures of representative tumors taken at day 11 exemplify the dramatic impact of cavitation-mediated enhanced VV delivery on tumor size (**Figure 4b**).

Survival analysis demonstrated that while 50% of all other groups were sacrificed before day 33 or sooner due to tumor size reaching the limit permitted under the license governing these studies, 50% of mice treated with cups + VVTK-RR-/FCU1 + US + 5-FC were not sacrificed until day 39 (see **Supplementary Table S1**).

It is notable that an impact on tumor retardation was evident in this experiment even prior to the commencement of 5-FC delivery. This does not directly align with the findings of **Figure 3** which demonstrates that although growth is slower than in controls at these early time-points with VVluc + cups + US, the effect is not as marked as that achieved with 10-fold lower titer of VVTK-RR-/FCU1 + cups + US in **Figure 4a**. It is hypothesized that the discrepancy in level of antitumor effect achieved with VVluc compared with VVTK-RR-/FCU1 may be due to differences in oncolytic efficacy of these two viruses in this HepG2 cell line.

## Discussion

Instigation of US mediated cavitation offers a noninvasive, safe, targetable and monitorable means of delivering and activating drugs within tumors.^10,13,22^ The inefficient delivery of OVs into and throughout tumors following their IV injection is one of the few remaining barriers to their widespread clinical translation.^23^ Interactions with complement, blood cells and the reticuloendothelial system very rapidly reduce the active circulating OV dose,^6,7^ while the high pressure and dense extracellular matrix within tumors restricts OV infection to the perivascular space and prevents optimal spread beyond initial infection foci.^10,24^ Hence, although OVs have now been approved for use in humans, this is in the context of intratumoral injection,^25,26^ a route which restricts potential efficacy^5^ and the range of applicable indications.

VV is a popular candidate for development as an OV due to its strong safety track record, well defined genome and large coding capacity. It is clear from clinical trials utilizing direct intratumoral delivery that the survival duration of patients is related to the VV dose delivered into their tumors.^27^ However, when injected systemically VV is prone to the bloodstream clearance mechanisms outlined above. This means that despite early promise,^28^ IV dosing of VV has not yet demonstrated marked clinical efficacy. Indeed, although a “breakthrough” dose of VV of 1.5 × 10^7^ pfu/kg has been identified, simply increasing the dose to enhance efficacy is not feasible in terms of cost or safety.^7^ It is clear that, although the VV dose remaining in the circulation following IV delivery may represent only a small percentage of that injected, it is still active and infective.^27,29,30^ Mechanisms of ensuring improved delivery of this active circulating dose into and throughout the tumor may offer a means by which clinical efficacy may be more readily achieved. While arming the vector with therapeutically powerful transgenes will ensure any VV which does successfully deposit, has as much antitumor effect as possible. We have previously demonstrated that technologies which instigate, control, and measure inertial cavitation can provide a powerful and targeted method to drive drugs, such as antibodies and oncolytic adenoviruses, deep into tumors following either intratumoral or intravenous delivery.^9,13,31^ Here we demonstrate this technology is well suited to delivery of oncolytic VV, with 1,000 to 10,000-fold increases in the infection of human cancer cell line xenografts in murine models, achieved only when VV is injected in combination with our proprietary cups formulation and US is focused on the tumor. Furthermore, use of this approach to deliver an oncolytic VV expressing an enzyme for the conversion of a prodrug into a cytotoxic metabolite adds further anticancer potency.^20,32^ Refined dosing with the prodrug 5-FC, which is converted into the active cytotoxic metabolite 5-FU, allows inhibition of cancer cell division without impacting too detrimentally on the replication of the VV.^32^ Hence, the enhanced delivery of a more effective armed VV, resulted in significant (*P* < 0.05) retardation of tumor growth following dosing with a single IV injection of just 100,000 copies of VVTK-RR-/FCU1. This is substantially below levels of VV vectors used in previous work where doses of 1 x 10^8^ VV (ref. 32) or two doses of 1 x 10^6^ VV (ref. 21) have been required to show efficacy.

Although this is an encouraging first demonstration of the combination of US technology and VV and an important step forward, it is clear that the antitumor efficacy does not yet match the level of VV delivery enhancement achieved. It is possible that the oncolytic virus “infection void” problem characterized by Miller *et al.*^23^ is still an important factor despite improved initial delivery. Experiments to investigate multiple cup and US treatment post-VV dosing to enhance spread from existing infection foci is an approach which will be studied in the further developments of this strategy.

We describe a clinically translatable technology, which does not require VV reformulation or surgically invasive procedures, but can enhance delivery and replication of the VV to such an extent that 10,000-fold enhancements of transgene expression can be achieved and therapeutic benefit can be detected following a single intravenous dose of just 100,000 copies. Recent studies have emphasized the challenge faced in achieving systemic delivery of vaccinia vectors and revealed interesting approaches to extend bloodstream circulation.^7^ This technology described here offers a targetable, safe, noninvasive means of ensuring that the active dose remaining in the circulation has the best chance possible of achieving antitumor efficacy.

## Materials and Methods

***Vaccinia viruses.*** Attenuated recombinant VVs were derived from the Copenhagen strain and were deleted in the thymidine kinase and ribonucleotide reductase genes. VVluc and VVTK-RR-/FCU1 expressed *Renilla* luciferase and FCU1, respectively. Viruses were propagated and titrated in chicken embryo fibroblasts as previously described in ref. 21.

***Cell lines.*** CT-26, HepG2, and SKOV-3 were obtained from the European Collection of Authenticated Cell Cultures (ECACC) and maintained according to their guidelines. For *in vitro* validation of VVluc activity, CT-26 cells at 10,000 cells/well in 96 well plates were exposed to 1 pfu VV per cell. VV had been exposed to cups + US or not. Cells were assayed for luciferase expression at 24 hours using Promega luciferase assay kit and a FLUOstar Omega microplate reader (BMG Labtech, Offenburg, Germany). A BCA assay was used to assess and standardize luciferase levels to protein concentration per well as described in reference 33.

***Cups manufacture.*** Cups manufacture was as described in reference 13. Following air drying to entrap air as nuclei for cavitation induction, cups were resuspended in sterile filtered 5% glucose solution to a concentration of 25 mg/ml and stored in a sterile rubber stoppered glass vial at room temperature. i-cups were not air dried before the resuspension in 5% glucose, but still matched the size, polydispersity, and surface charge of active air dried cups.

***US equipment and parameters.*** US set-up and exposure parameters were as described in ref. 10 except that the system utilizes a linear array diagnostic US probe (instead of a single-element passive cavitation detector) and US generation/reception platform for conducting real-time B-mode imaging, therapeutic US transmit and real-time treatment monitoring by passive acoustic mapping.

**In vivo *studies.*** UK Home Office guidelines and the UKCCCR Guidelines for the Welfare of Animals in Experimental Neoplasia were followed. CT-26 cells (100 µl containing 2 × 10^5^) were implanted into the flanks of BALB/c nude mice using a 27 gauge needle. HepG2 or SKOV-3 cells (100 µl containing 5 × 10^6^) were implanted in a 1:1 mix of matrigel into the flanks of CD1 nude mice using a 27 gauge needle. When CT-26 tumors had reached 200–500 mm^3^ or HepG2 and SKOV-3 tumors had reached 40–100 mm^3^, mice were randomized into treatment groups and treated according to a protocol whereby the focus of a 0.5 MHz transducer was aligned onto the tumor using a B-mode image captured using a L11-4 linear array probe. Tumors were exposed to US (1.5 MPa peak negative focal pressure, 500 kHz driving frequency, 0.5 Hz pulse repetition frequency, and 5% duty cycle) and, provided no cavitation signal from within the tumor was detected, 50 µl of VV or cups + VV (final concentration of VV as stated in figure legends, final concentration of cups = 25 mg/ml) was injected via a cannula into the tail vein 10 seconds later. A dose of 25 mg/ml was used as a result of studies that demonstrated that this level gave the highest and most reliable level of cavitation (see **Supplementary Figure S6**). This dose did not induce toxicity in these or previous studies^13^ and increased the likelihood of VV and cup colocalization within tumor vasculature. At 2 minutes the focus of the US was moved to a different point within the tumor and at 4 minutes a further injection of 50 µl of cups + VV was administered. Over 10 minutes US exposure continued with further movement of the focus within the tumor at 6 and 8 minutes. Passive acoustic mapping was as described in ref. 34. 5-FC dosing was performed by daily s.c. injection of 100 µl of a 12.5 mg/ml solution 7 days after IV treatment. CT-26 studies were performed as described for HepG2 studies but mice were sacrificed, tumors lysed and luciferase expression measured 48 hours after treatment.

***Tracking of delivery and therapy.*** Delivery was assessed by IVIS imaging as described in ref. 10. Replication of the VV was assessed by performing quantitative polymerase chain reaction for VV genomes at 20 days after treatment. Tumors and organs were homogenized using a mechanical disruptor and DNA isolated as in ref. 9. Primer sequences AGATCATCGTATGGAGAGTCGTAAGAT and TGACTACGTTGTTATGAGTGCTTGGTA and probe sequence [6FAM]ATCAAAATACAAGAC

GTCGCTTTTAGCAGCTAAAAGAA[TAM] (Sigma, Welwyn Garden City, UK) were used at 100 µmol/l and 10 µmol/l respectively with a quantitative polymerase chain reaction Bio Probe mastermix with Rox (PCR Biosystems, London, UK) reference standard according to the manufacturer's instructions. A standard curve of known VV concentrations spiked into tumor or organ lysates and DNA extracted was run to quantify the number of VV genome copies. Tumor growth was tracked using caliper measurements and the equation h × w × l / 2.

**SUPPLEMENTARY MATERIAL** **Figure S1**. *In vitro* infectivity of vaccinia virus after exposure to cavitation. **Figure S2.** Passive acoustic mapping of cup nucleated US mediated cavitation from within ultrasound exposed tumors. **Figure S3.** VV genome copy number within the livers of mice at sacrifice on day 20. **Figure S4.** *In vivo* infectivity of vaccinia virus delivered using polymeric cup (“cups”) nucleated cavitation to SKOV-3 tumors. **Figure S5.** SKOV-3 and HepG2 tumor growth in mice dosed IV with 1 × 10^5^ copies of VVluc. **Figure S6.** Influence of cup concentration on cavitation signal from within focal region in tumor. **Table S1.** Median survival of mice dosed with VVTK-RR-/FCU1 and 5-Fluorocytosine (5-FC).
