# Supplementary material for: Polymeric Cups for Cavitation-mediated Delivery of Oncolytic Vaccinia Virus
Source: Mol Ther. 2016 Aug 9;24(9):1627–33. doi: 10.1038/mt.2016.139 (PMC5113106; doi:10.1038/mt.2016.139)
Supplement: Supplementary Figures and Table [file mt2016139x1.ppt]

## Slide 1
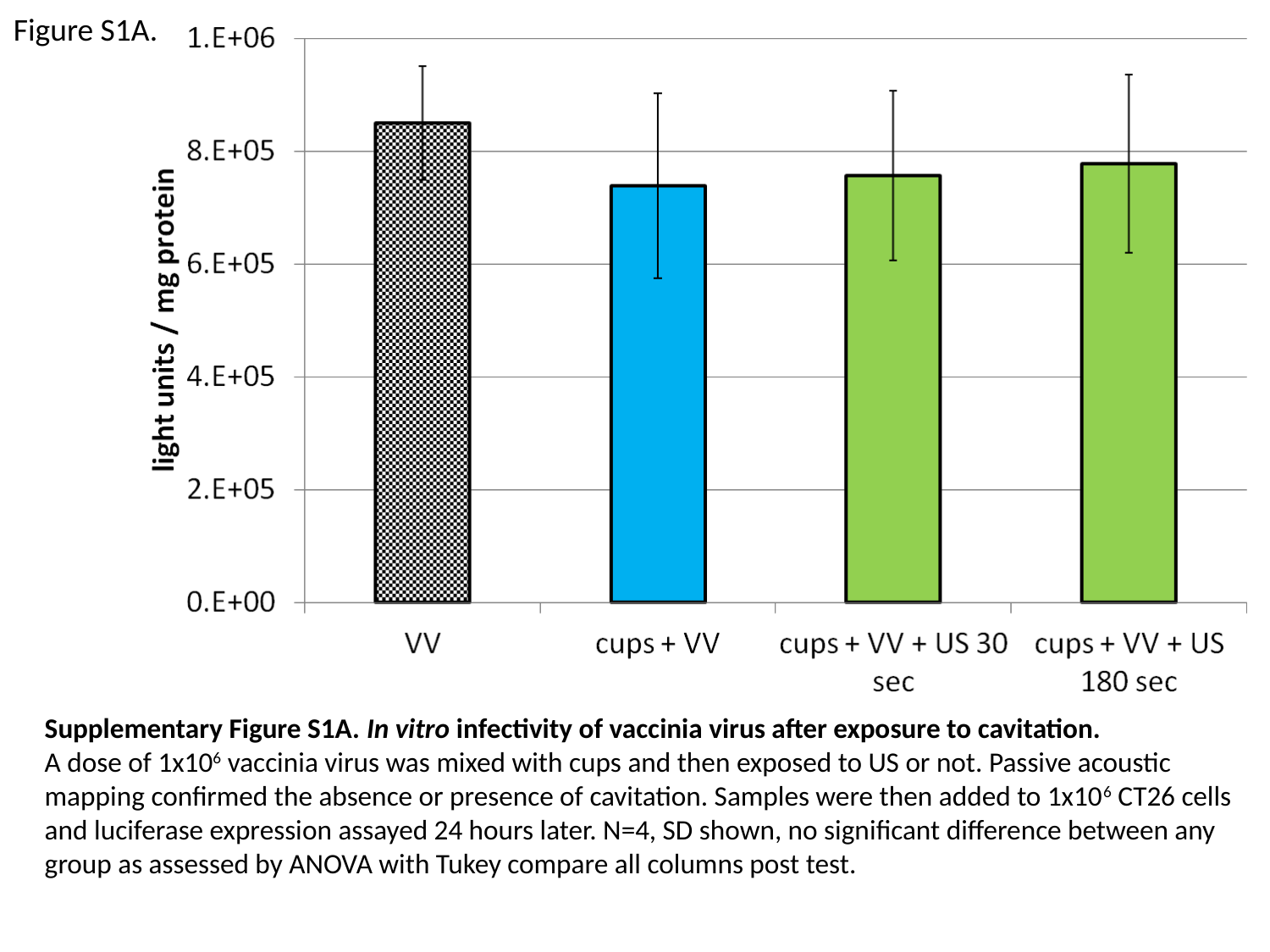

Figure S1A.
Supplementary Figure S1A. In vitro infectivity of vaccinia virus after exposure to cavitation.
A dose of 1x10^6^ vaccinia virus was mixed with cups and then exposed to US or not. Passive acoustic mapping confirmed the absence or presence of cavitation. Samples were then added to 1x10^6^ CT26 cells and luciferase expression assayed 24 hours later. N=4, SD shown, no significant difference between any group as assessed by ANOVA with Tukey compare all columns post test.

## Slide 2
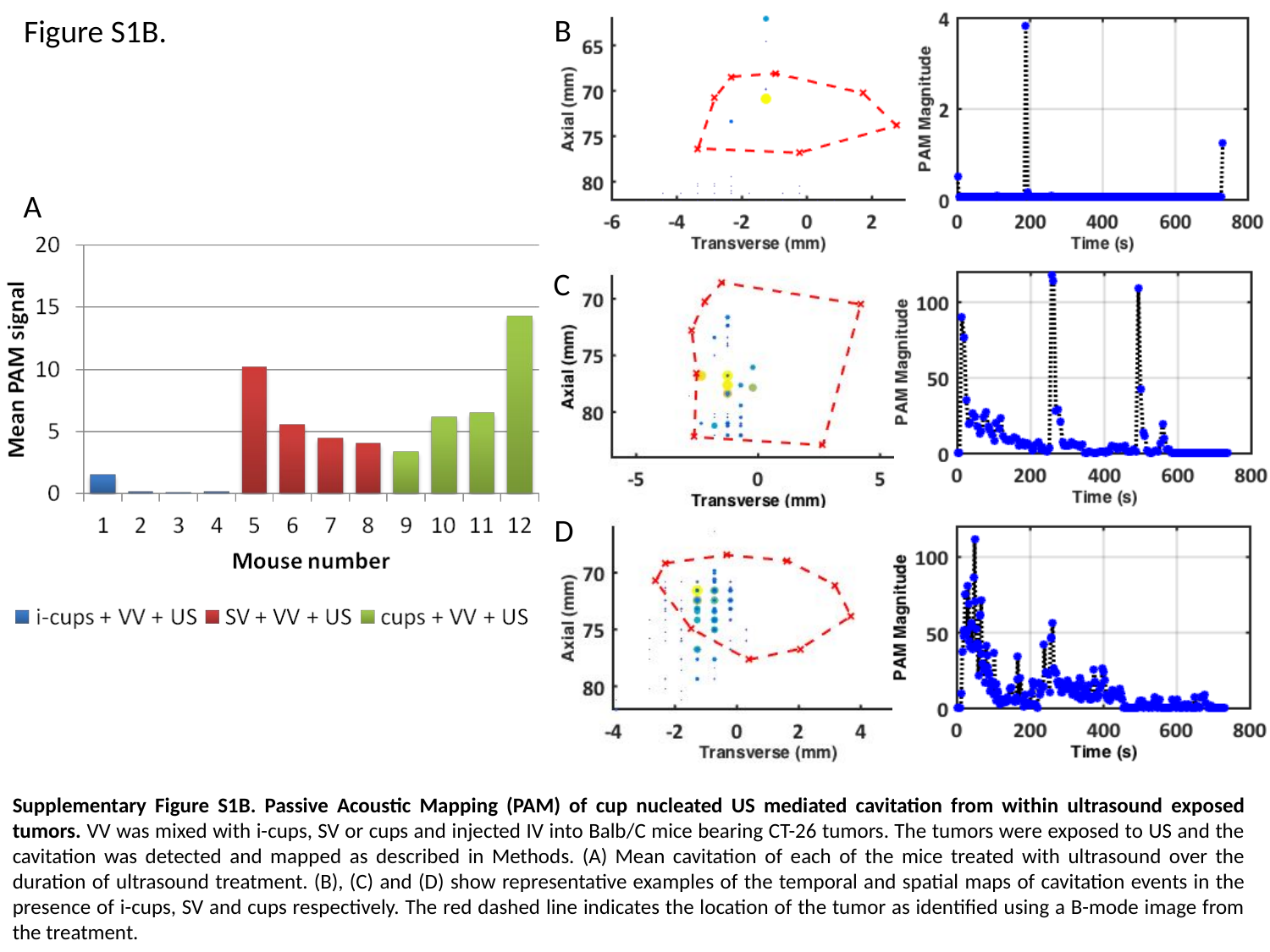

B
C
D
A
Figure S1B.
Supplementary Figure S1B. Passive Acoustic Mapping (PAM) of cup nucleated US mediated cavitation from within ultrasound exposed tumors. VV was mixed with i-cups, SV or cups and injected IV into Balb/C mice bearing CT-26 tumors. The tumors were exposed to US and the cavitation was detected and mapped as described in Methods. (A) Mean cavitation of each of the mice treated with ultrasound over the duration of ultrasound treatment. (B), (C) and (D) show representative examples of the temporal and spatial maps of cavitation events in the presence of i-cups, SV and cups respectively. The red dashed line indicates the location of the tumor as identified using a B-mode image from the treatment.

## Slide 3
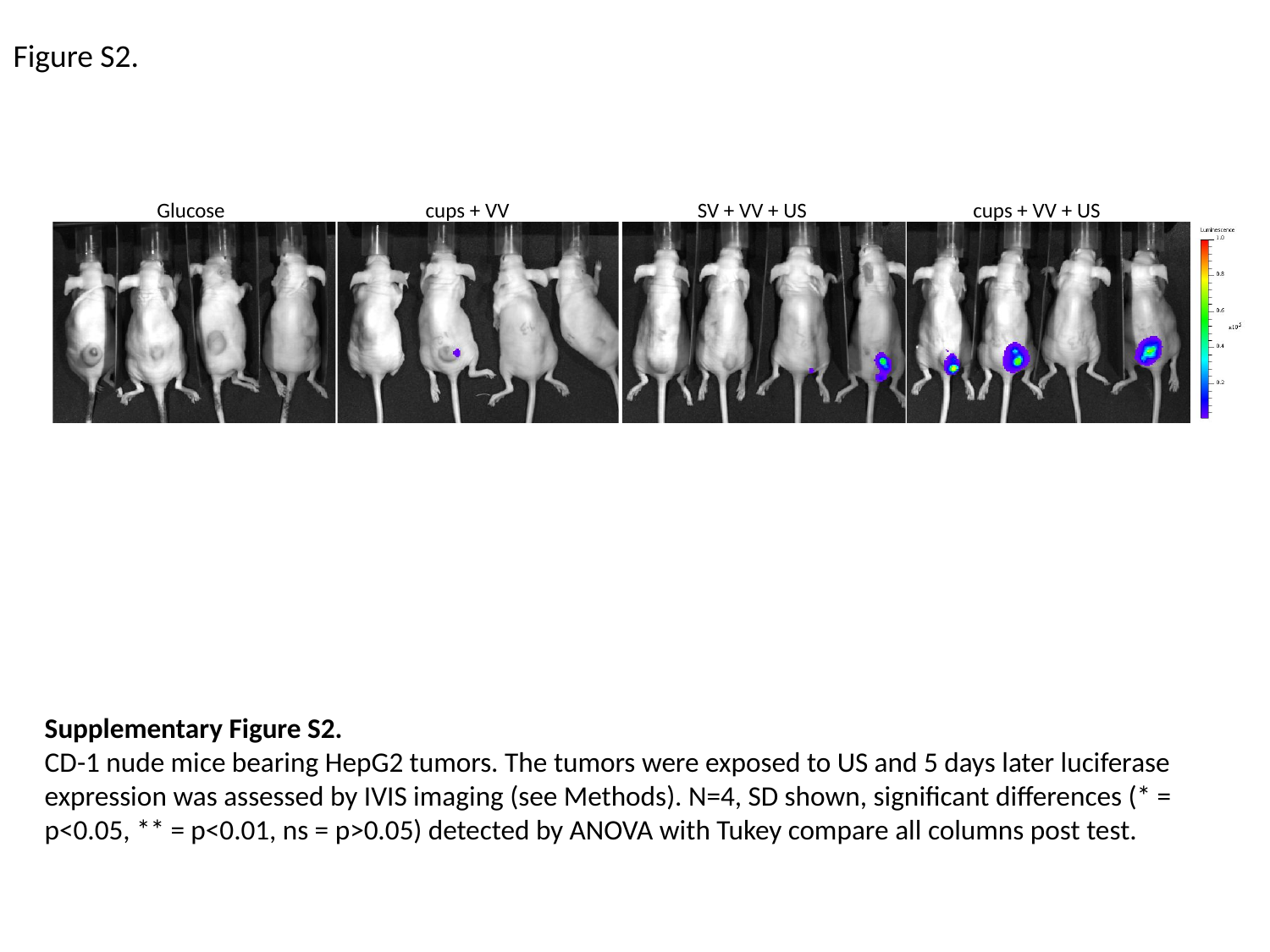

Figure S2.
Glucose
cups + VV
SV + VV + US
cups + VV + US
Supplementary Figure S2.
CD-1 nude mice bearing HepG2 tumors. The tumors were exposed to US and 5 days later luciferase expression was assessed by IVIS imaging (see Methods). N=4, SD shown, significant differences (* = p<0.05, ** = p<0.01, ns = p>0.05) detected by ANOVA with Tukey compare all columns post test.

## Slide 4
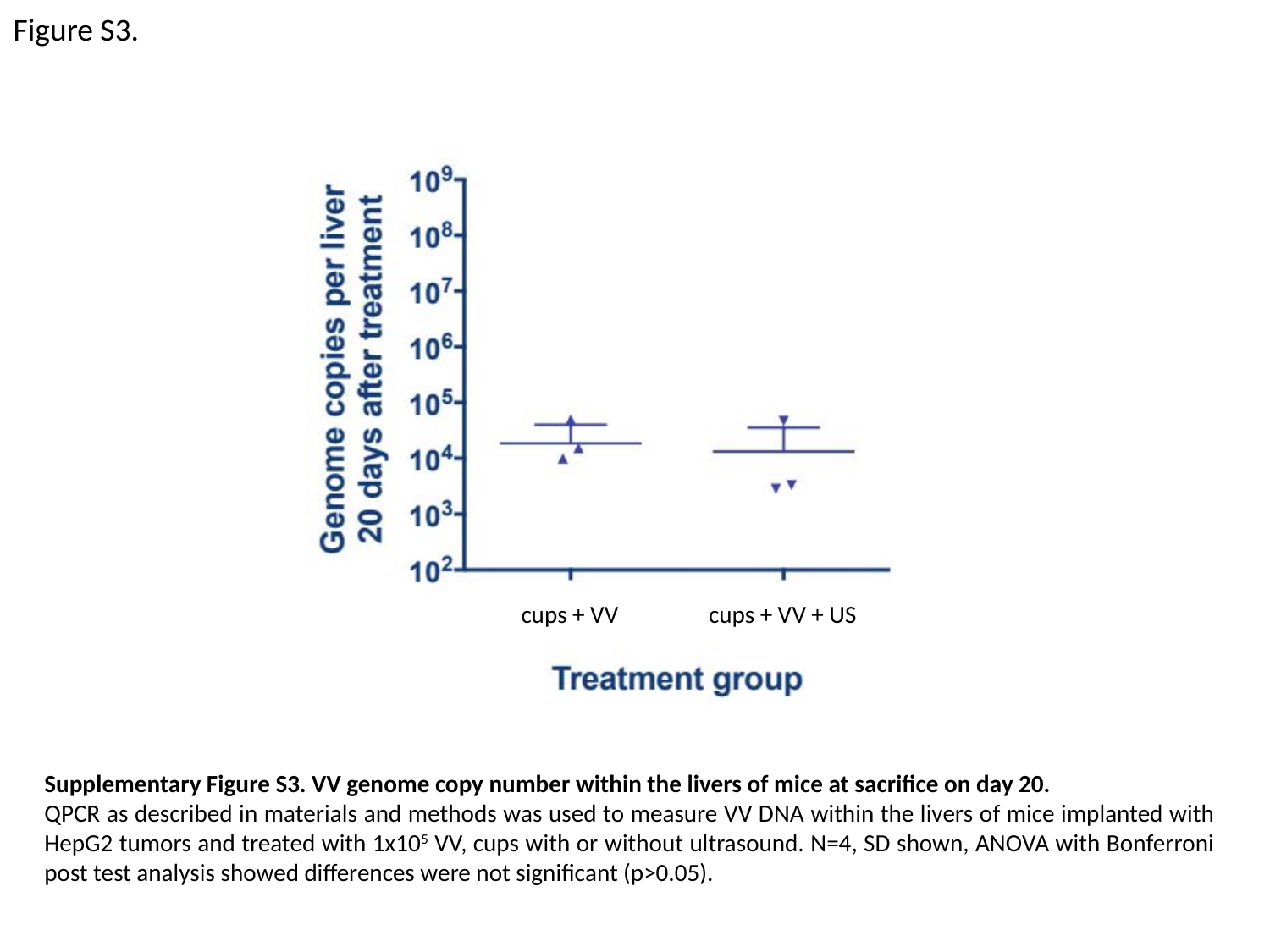

Figure S3.
 cups + VV	 cups + VV + US
Supplementary Figure S3. VV genome copy number within the livers of mice at sacrifice on day 20.
QPCR as described in materials and methods was used to measure VV DNA within the livers of mice implanted with HepG2 tumors and treated with 1x10^5^ VV, cups with or without ultrasound. N=4, SD shown, ANOVA with Bonferroni post test analysis showed differences were not significant (p>0.05).

## Slide 5
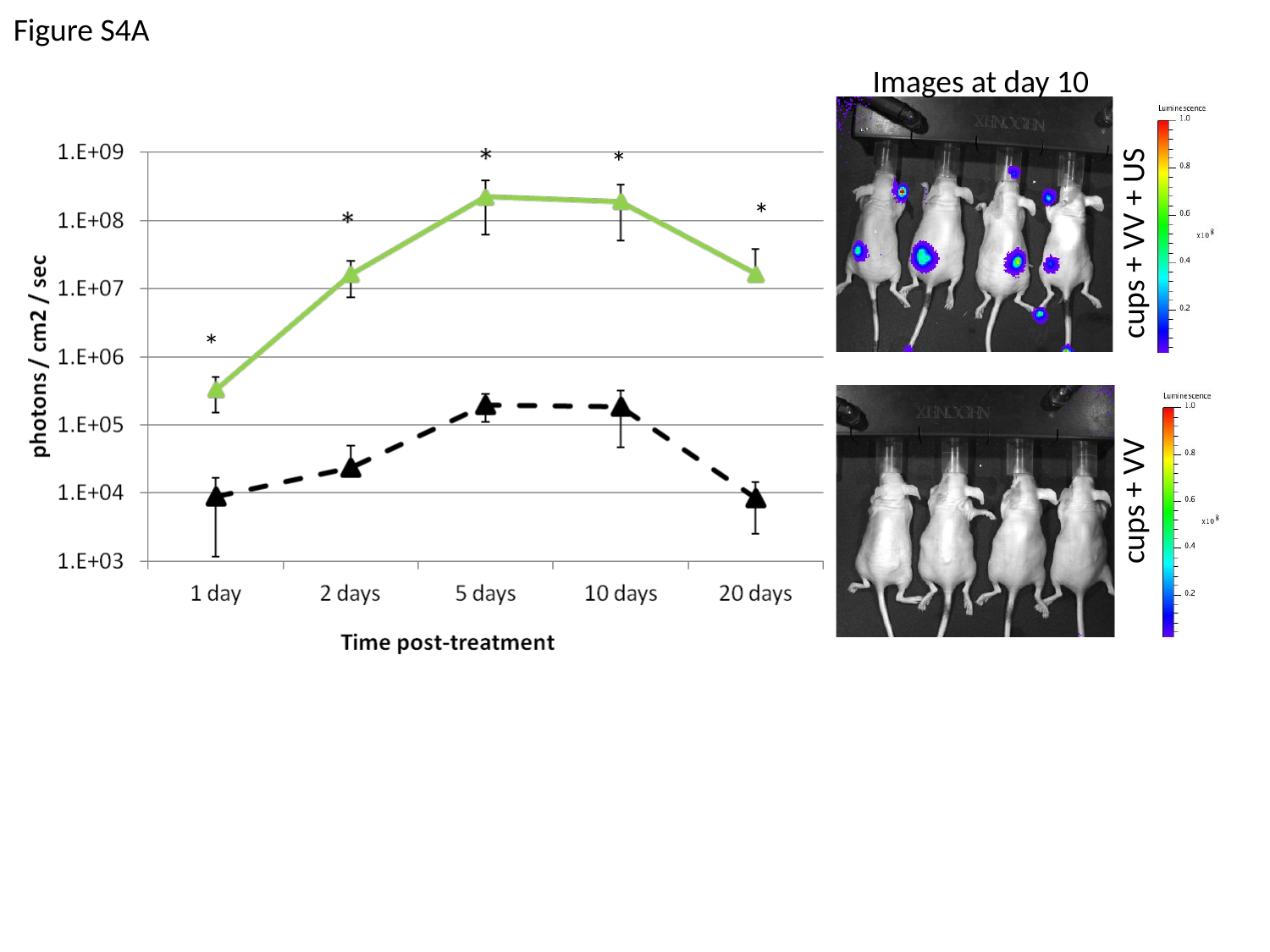

Figure S4A
Images at day 10
*
*
*
 cups + VV 	cups + VV + US

## Slide 6
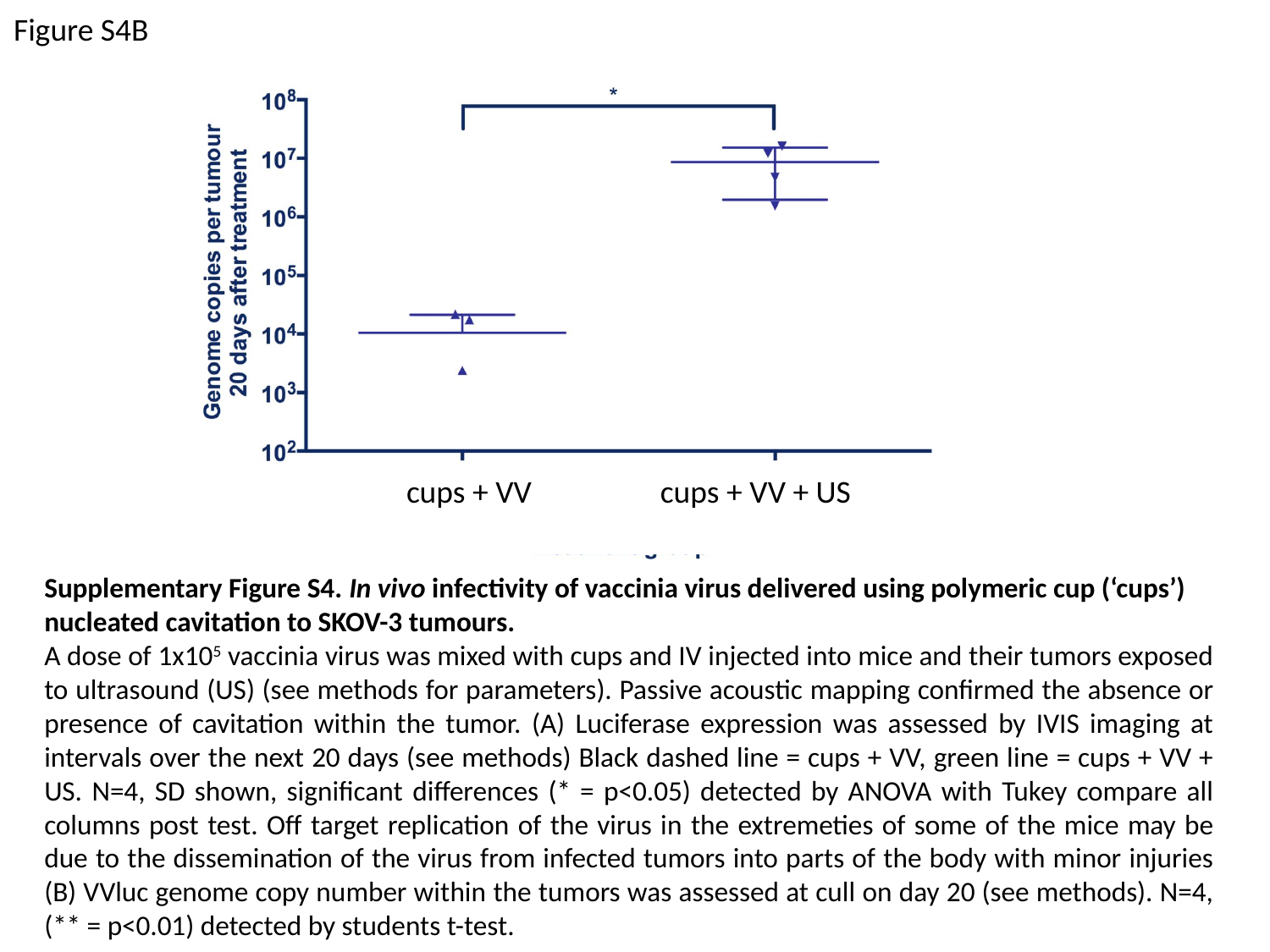

Figure S4B
 cups + VV 	 cups + VV + US
Supplementary Figure S4. In vivo infectivity of vaccinia virus delivered using polymeric cup (‘cups’) nucleated cavitation to SKOV-3 tumours.
A dose of 1x10^5^ vaccinia virus was mixed with cups and IV injected into mice and their tumors exposed to ultrasound (US) (see methods for parameters). Passive acoustic mapping confirmed the absence or presence of cavitation within the tumor. (A) Luciferase expression was assessed by IVIS imaging at intervals over the next 20 days (see methods) Black dashed line = cups + VV, green line = cups + VV + US. N=4, SD shown, significant differences (* = p<0.05) detected by ANOVA with Tukey compare all columns post test. Off target replication of the virus in the extremeties of some of the mice may be due to the dissemination of the virus from infected tumors into parts of the body with minor injuries (B) VVluc genome copy number within the tumors was assessed at cull on day 20 (see methods). N=4, (** = p<0.01) detected by students t-test.

## Slide 7
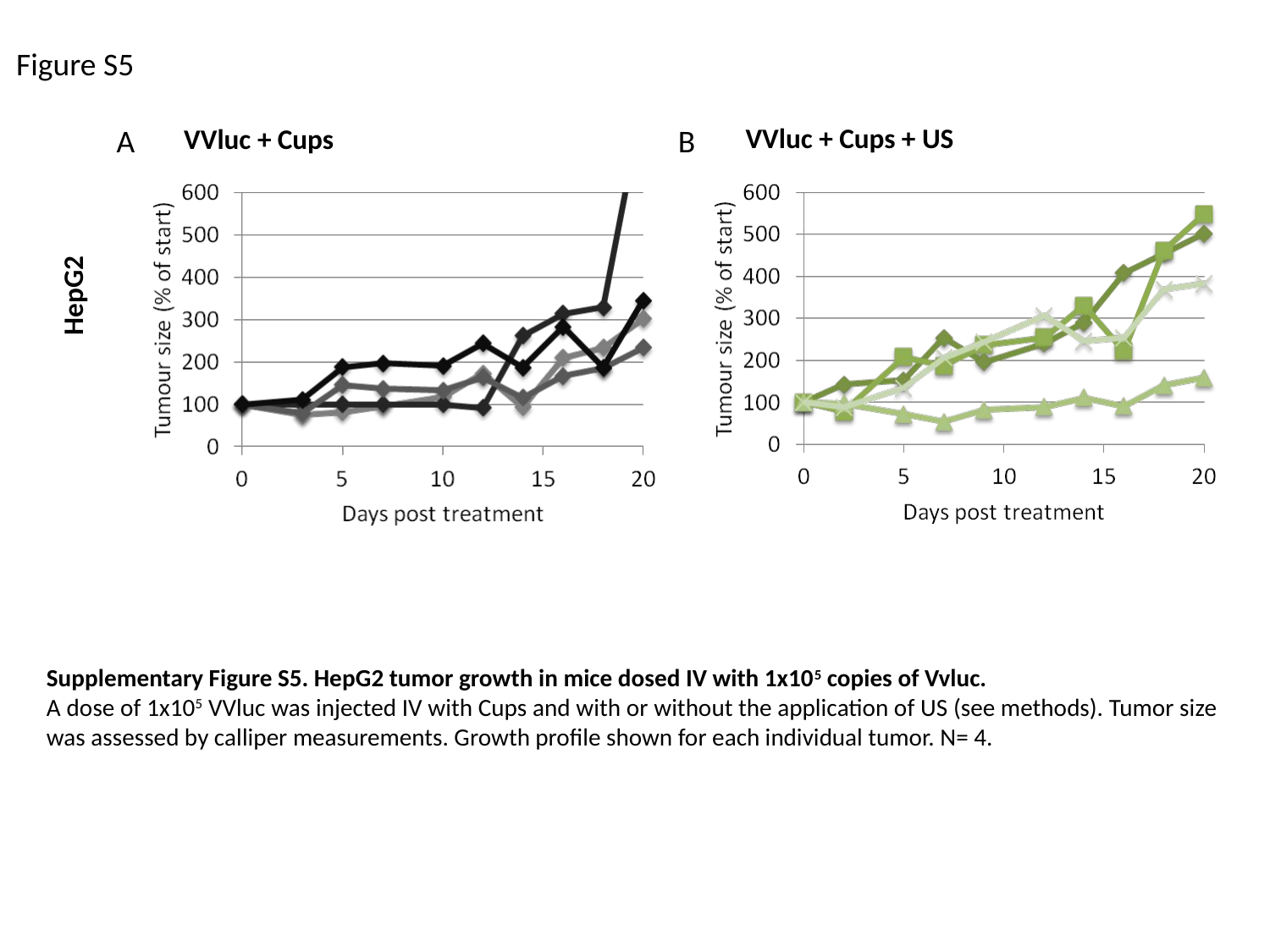

Figure S5
VVluc + Cups + US
A
VVluc + Cups
B
HepG2
Supplementary Figure S5. HepG2 tumor growth in mice dosed IV with 1x10^5^ copies of Vvluc.
A dose of 1x10^5^ VVluc was injected IV with Cups and with or without the application of US (see methods). Tumor size was assessed by calliper measurements. Growth profile shown for each individual tumor. N= 4.

## Slide 8
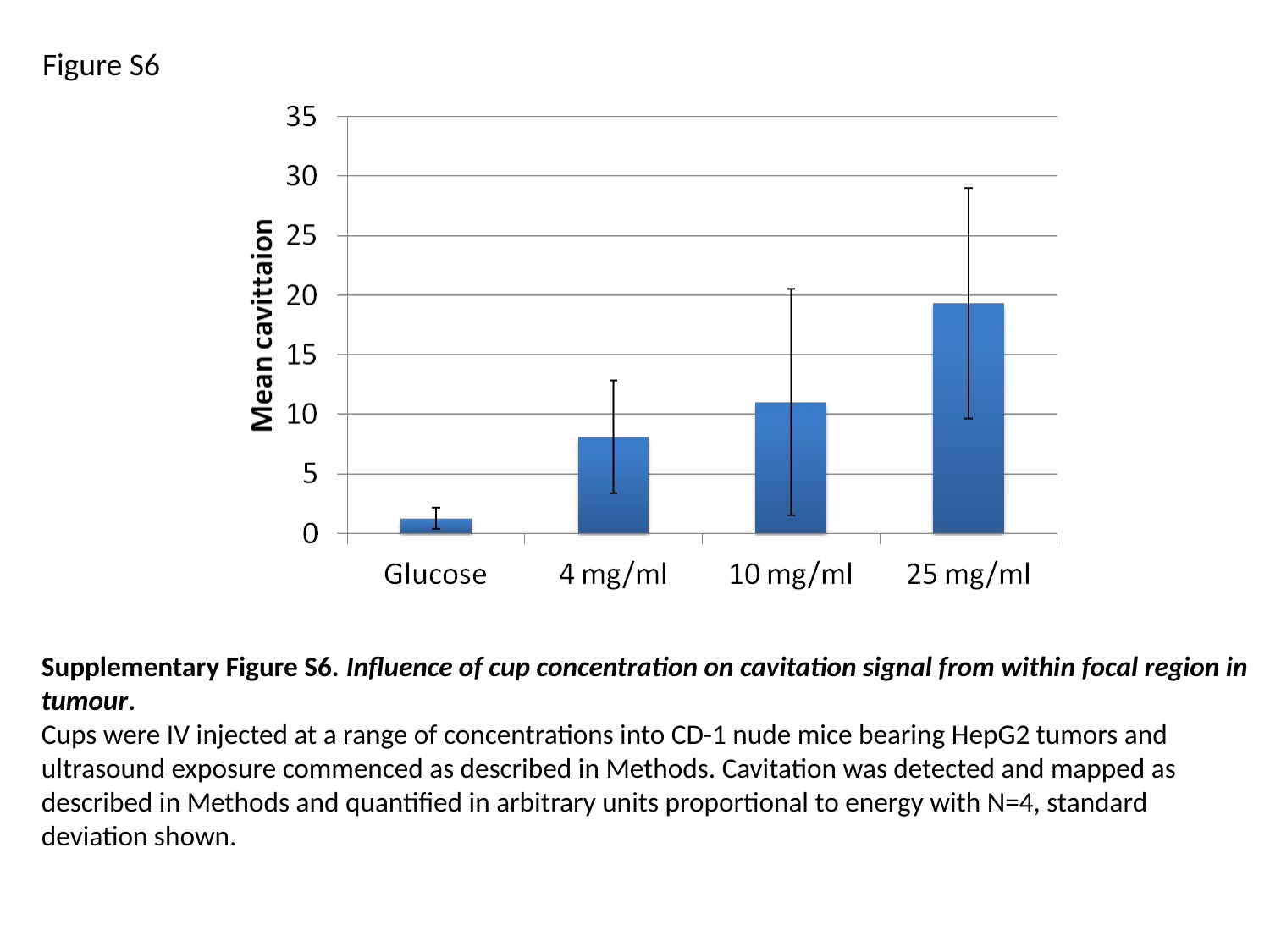

Figure S6
Supplementary Figure S6. Influence of cup concentration on cavitation signal from within focal region in tumour.
Cups were IV injected at a range of concentrations into CD-1 nude mice bearing HepG2 tumors and ultrasound exposure commenced as described in Methods. Cavitation was detected and mapped as described in Methods and quantified in arbitrary units proportional to energy with N=4, standard deviation shown.

## Slide 9
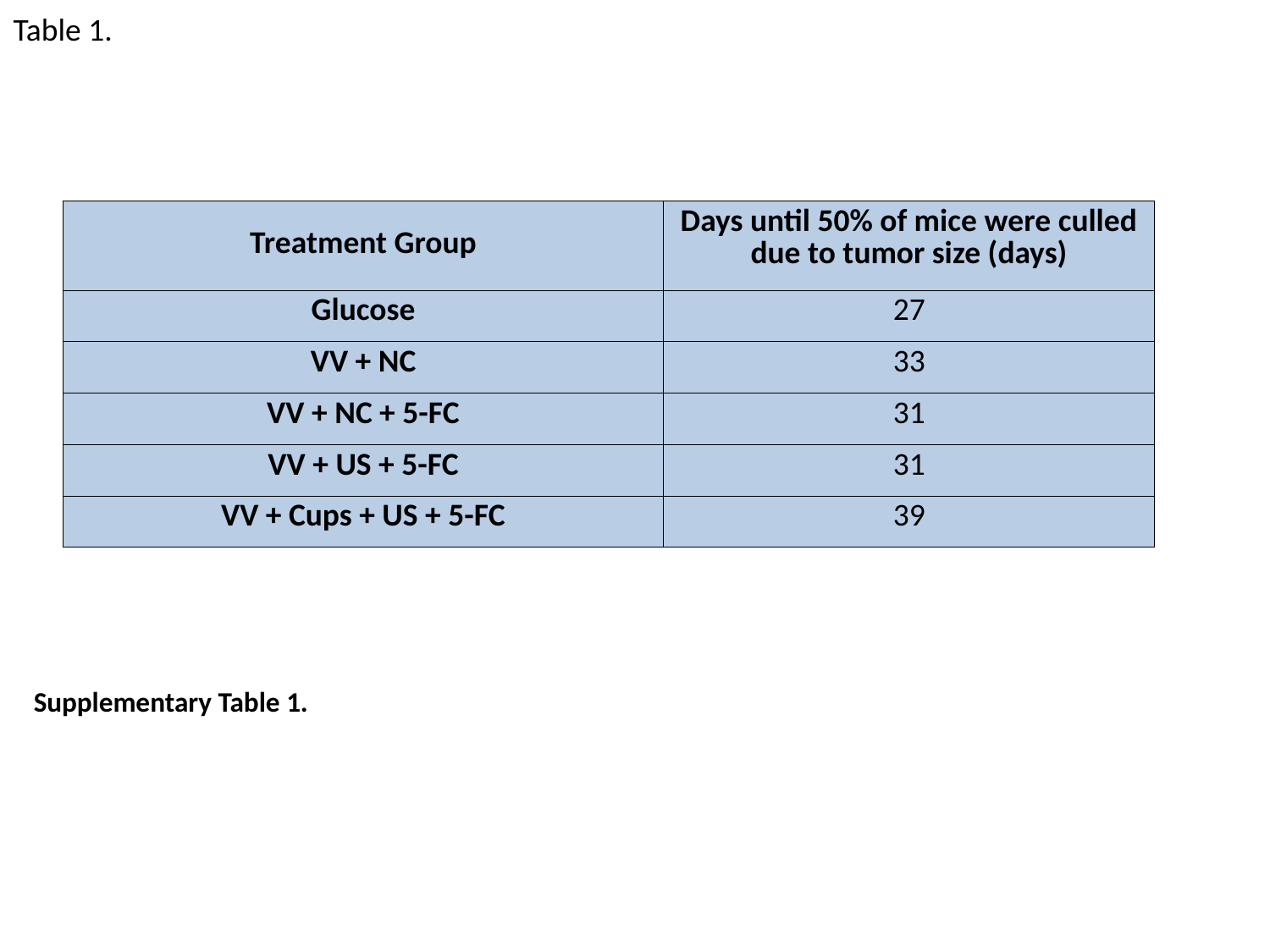

Table 1.
| Treatment Group | Days until 50% of mice were culled due to tumor size (days) |
| --- | --- |
| Glucose | 27 |
| VV + NC | 33 |
| VV + NC + 5-FC | 31 |
| VV + US + 5-FC | 31 |
| VV + Cups + US + 5-FC | 39 |
Supplementary Table 1.
